# Menopausal status, age and management among women living with HIV in the UK

**DOI:** 10.1111/hiv.13138

**Published:** 2021-07-26

**Authors:** Hajra Okhai, Caroline A. Sabin, Katharina Haag, Lorraine Sherr, Rageshri Dhairyawan, Fiona Burns, Richard Gilson, Frank Post, Jonathan Ross, Nicola Mackie, Ann Sullivan, Jane Shepherd, Anjum Tariq, Rachael Jones, Julie Fox, Melanie Rosenvinge, Shema Tariq

**Affiliations:** ^1^ Institute for Global Health University College London London UK; ^2^ Department of Infection and Immunity Barts Health NHS Trust London UK; ^3^ Royal Free London NHS Foundation Trust London UK; ^4^ King’s College Hospital NHS Foundation Trust London UK; ^5^ University Hospital Birmingham NHS Foundation Trust Birmingham UK; ^6^ Imperial College Healthcare NHS Trust London UK; ^7^ Chelsea and Westminster Healthcare NHS Foundation Trust London UK; ^8^ UK Community Advisory Board London UK; ^9^ The Royal Wolverhampton Hospitals NHS Trust Wolverhampton UK; ^10^ Lewisham and Greenwich NHS Trust London UK

**Keywords:** ageing, hormone replacement therapy, menopause, symptoms, women

## Abstract

**Background:**

There is currently little evidence exploring menopausal status, age at last menstrual period (LMP) and management of menopause among women living with HIV aged 45–60 years in England.

**Methods:**

Socio‐demographic, lifestyle and clinical data were collected through a self‐completed cross‐sectional survey. Longitudinal CD4 count and viral load data were available from linkage to clinical records, if consent was provided. Women were categorised as pre‐, peri‐ or post‐menopausal. Factors associated with menopausal stage were examined using ordinal logistic regression adjusting for age. Age at LMP was estimated using Kaplan–Meier survival analysis.

**Results:**

The 847 women had a median age of 49 [interquartile range (IQR): 47–52] years. Most were of black ethnicity (81.3%), were born outside the UK (85.0%) and had completed secondary education (88.7%); 177 (20.4%), 373 (43.0%) and 297 (34.2%) were pre‐, peri‐ or post‐menopausal, respectively. After adjusting for age, associations of menopausal status with non‐cohabiting relationship [adjusted odds ratio = 0.63 (95% confidence interval: 0.43–0.91)], baseline viral load ≥ 100 000 copies/mL [2.67 (1.20–5.94)] and unemployment [1.34 (0.97–1.84)] remained significant. Median (IQR) age at LMP was 54 (51–55) years in the group. In total, 27.9% (233/836) of women reported severe menopausal symptoms; 45.6% of those with somatic symptoms had heard of hormone replacement therapy and 8.7% had used it. Only 5.6% of women with urogenital symptoms had used topical oestrogen.

**Conclusions:**

Our findings highlight the importance of educating both women and their healthcare providers about menopausal symptoms and management options.

## INTRODUCTION

Menopause is a key life event for women, with the associated physical and psychological changes often having a significant impact on quality of life [1]. In the general population, natural menopause, defined as amenorrhoea for > 12 months as a result of diminishing ovarian reserve, generally occurs between the ages of 45 and 55 years (median age 51) [2]. Premature ovarian insufficiency (defined as menopause occurring at ages < 40 years) is associated with an increased risk of osteoporosis, cardiovascular disease (CVD) and poorer cognitive function [3, 4, 5, 6].

In the UK, one in three women living with HIV is now over the age of 50 [7]. With life expectancy reaching that of their HIV‐negative counterparts, women living with HIV are increasingly encountering age‐ and menopause‐related health concerns. The interplay between chronic HIV infection and oestrogen depletion is poorly understood, with few studies having explored menopause among women living with HIV [8].

The Positive Transitions Through the Menopause (PRIME) Study, a cross‐sectional, mixed‐methods observational study, was designed to explore the association between menopause, and the health and well‐being of women living with HIV in the UK [9]. In this analysis, we use PRIME Study data to describe characteristics of women with HIV in different stages of the menopause, to identify factors independently associated with this, and to establish the median age at last menstrual period (LMP). Finally, we explore the association between severity of menopausal symptoms and knowledge and use of hormonal replacement therapy (HRT) among women attending NHS HIV clinics in England.

### METHODS

The PRIME Study methods are described elsewhere [9]. In brief, women (defined based on sex assigned at birth) aged 45–60 years were recruited from 21 NHS Trusts that provide HIV services across England between February 2016 and June 2017. Women were ineligible if they had experienced surgical menopause, had received chemotherapy or radiotherapy in the last 6 months, had used hormonal contraception within the last 6 months for either contraceptive or non‐contraceptive use (women who had an intrauterine insertion when commencing HRT were included) or if their LMP occurred > 60 months prior to study enrolment. The PRIME Study has ethical approval from the South East Coast‐Surrey Research Ethics Committee (ref. 15/0735).

Self‐completed paper questionnaires collected information on: demographic/social factors (age, ethnicity, UK‐born, immigration, employment, relationship and financial status, educational attainment); comorbidities (hepatitis B/C, hypertension, diabetes, CVD, stroke, osteoporosis, breast cancer); current lifestyle [smoking, recreational drug use, alcohol use (assessed using the Alcohol Use Disorders Identification Test, AUDIT‐C, with a score ≥ 5 considered to be harmful)]; HIV history (years since HIV diagnosis, most recent CD4 count and HIV viral load); menopause‐related symptoms [assessed using the Menopause Rating Scale (MRS) [10]] and care‐seeking behaviour (knowledge and use of hormone therapies).

The total number of medical conditions reported by women was used as a continuous variable to explore the impact of multi‐morbidity. For women who consented, questionnaire data were supplemented with routinely collected HIV data from clinical records, including nadir CD4 count and baseline viral load (first available viral load measure following HIV diagnosis).

Menopausal status was determined from self‐reported menstrual pattern (without biological confirmation) and categorized as pre‐menopausal (regular menstruation), peri‐menopausal (irregular periods over the previous 2 years) and post‐menopausal (amenorrhoea for 12 months or more). For the present analyses, we excluded 21 women in whom menopausal status could not be established.

Similar to menopausal status, age at LMP was estimated for post‐menopausal women based on self‐reported menstrual pattern. We estimated age at LMP as the woman's age at questionnaire completion minus 1 year for those who reported that they had last had a period 1–2 years before the study, or as age at questionnaire completion minus 3 years for those who reported that they had last had a period 2–5 years before the study.

Characteristics at the time of questionnaire completion were compared across the three menopausal stages using χ^2^ and Kruskal–Wallis tests, as appropriate. Factors associated with menopausal status were assessed using an ordinal logistic regression model, due to the ordered nature of menopausal status. Therefore, assuming the underlying effect of each covariate for those in the peri‐ *vs*. pre‐menopausal groups was the same as that for those in the post‐ *vs*. peri‐menopausal groups, allowing a common odds ratio (OR) to be estimated. Analyses of associations of each factor with menopausal status included adjustment for age given the strong expected correlation between age and menopausal status, and the known associations of many of these factors with age.

We estimated the median age at LMP in the study among women at all menopausal stages using a survival analysis approach, with age considered as the time axis. For women in the post‐menopausal group, the ‘follow‐up time’ was set to the age at LMP; ‘follow‐up times’ for those in the pre‐ and peri‐menopausal groups were right‐censored at age at questionnaire completion. ‘Follow‐up’ times on women were additionally left‐censored at the age of HIV diagnosis, thus effectively excluding women who were diagnosed with HIV after LMP from the analysis as the temporal associations were unclear. The median age at LMP was estimated using the Kaplan–Meier method.

Finally, we described the severity of menopausal symptoms and their association with menopausal stage and use of HRT or topical oestrogen. The MRS questionnaire comprises 11 symptoms grouped into three domains (somatic, psychological and urogenital). Women reported the perceived severity of each symptom on a scale of 0 (no symptoms) to 4 (severe symptoms). Overall and domain‐specific composite scores were generated by summing individual scores (see Appendix 1).

### RESULTS

Of the 1312 eligible women living with HIV who were approached, 80.7% (1058/1312) consented to participate, and 66.2% (868/1312) had complete questionnaires available. Of these, menopausal status could be established for 847 women who were included in the present analyses (Table 1).

**TABLE 1:** Demographic, social and clinical characteristics relating to HIV and menopause reported by women who completed the PRIME Study questionnaire, stratified by menopausal status

Question	All	Pre‐ menopausal	Peri‐menopausal	Post‐menopausal	*P*‐value
Total	847	177	373	297	
Age at completion of questionnaire [median (IQR)] (years)	49 (47–52)	47.0 (46.0–49.0)	49.0 (47.0–51.0)	53.0 (50.0–55.0)	< 0.001
Ethnicity					
Black African	589 (71.8%)	133 (76.9%)	258 (70.9%)	198 (70.0%)	0.34
White	103 (12.6%)	12 (6.9%)	52 (14.3%)	39 (13.8%)	
Black other	78 (9.5%)	18 (10.4%)	32 (8.8%)	28 (9.9%)	
Other	50 (6.1%)	10 (5.8%)	22 (6.0%)	18 (6.4%)	
Not born in UK	709 (85.0%)	161 (92.0%)	313 (84.8%)	235 (81.0%)	0.006
Insecure immigration status (where response to born in UK was ‘no’)	33 (4.8%)	8 (5.1%)	12 (4.0%)	13 (5.8%)	0.63
Employment status					
Full‐time employment	401 (49.3%)	89 (51.4%)	182 (50.4%)	130 (46.6%)	0.093
Part‐time employment	140 (17.2%)	36 (20.8%)	64 (17.7%)	40 (14.3%)	
No employment	272 (33.5%)	48 (27.7%)	115 (31.9%)	109 (39.1%)	
Are you currently in an ongoing relationship?					
No	374 (47.0%)	68 (41.5%)	147 (41.9%)	159 (56.8%)	< 0.001
Non‐cohabiting	161 (20.3%)	35 (21.3%)	85 (24.2%)	41 (14.6%)	
Cohabiting	260 (32.7%)	61 (37.2%)	119 (33.9%)	80 (28.6%)	
Completed education					
Did not finish school	90 (11.3%)	17 (10.2%)	38 (10.7%)	35 (12.8%)	0.75
High school	351 (44.2%)	70 (42.2%)	157 (44.2%)	124 (45.4%)	
University	353 (44.5%)	79 (47.6%)	160 (45.1%)	114 (41.8%)	
Do you currently have enough money to cover your basic needs?					
All of the time	308 (36.8%)	65 (36.9%)	144 (39.3%)	99 (33.7%)	0.47
Most of the time	221 (26.4%)	40 (22.7%)	99 (27.0%)	82 (27.9%)	
Some of the time	219 (26.2%)	49 (27.8%)	92 (25.1%)	78 (26.5%)	
No	88 (10.5%)	22 (12.5%)	31 (8.5%)	35 (11.9%)	
Current smoker	69 (8.4%)	9 (5.2%)	36 (10.0%)	24 (8.2%)	0.17
Recreational drug use in past 3 months	21 (2.5%)	3 (1.7%)	9 (2.4%)	9 (3.2%)	0.63
Harmful alcohol use (AUDIT‐C score)					
No alcohol use	332 (39.2%)	74 (45.7%)	134 (38.3%)	124 (41.8%)	0.54
< 5	387 (45.7%)	77 (47.5%)	181 (51.7%)	129 (43.4%)	
≥ 5	128 (15.1%)	11 (6.8%)	35 (10.0%)	44 (14.8%)	
Total number of medical conditions reported					
0	544 (64.2%)	131 (74.0%)	241 (64.6%)	172 (57.9%)	0.003
1	229 (27.0%)	38 (21.5%)	104 (27.9%)	87 (29.3%)	
2	63 (7.4%)	8 (4.5%)	25 (6.7%)	30 (10.1%)	
≥ 3	11 (1.3%)	0 (0.0%)	3 (0.8%)	8 (2.7%)	
Last HIV viral load (copies/mL)					
Undetectable	703 (88.2%)	146 (89.0%)	318 (87.6%)	239 (88.5%)	0.88
Detectable	94 (11.8%)	18 (11.0%)	45 (12.4%)	31 (11.5%)	
Baseline HIV viral load (copies/mL)					
Undetectable	31 (7.8%)	9 (13.0%)	14 (7.9%)	8 (5.3%)	0.055
< 10 000	106 (26.6%)	22 (31.9%)	50 (28.2%)	34 (22.4%)	
10 000–99 999	131 (32.9%)	21 (30.4%)	63 (35.6%)	47 (30.9%)	
≥ 100 000	130 (32.7%)	17 (24.6%)	50 (28.2%)	63 (41.4%)	
Lowest (nadir) CD4 count (cells/μL)					
> 500	39 (7.2%)	6 (5.6%)	20 (8.0%)	13 (6.9%)	0.17
200–500	182 (33.4%)	45 (42.1%)	84 (33.6%)	53 (28.2%)	
< 200	324 (59.4%)	56 (52.3%)	146 (58.4%)	122 (64.9%)	
Last CD4 count (cells/μL)					
>500	512 (68.6%)	101 (66.9%)	246 (73.2%)	165 (63.7%)	0.13
200–500	187 (25.1%)	38 (25.2%)	72 (21.4%)	77 (29.7%)	
< 200	47 (6.3%)	12 (7.9%)	18 (5.4%)	17 (6.6%)	

IQR, interquartile range.

Results are *n* (%) unless otherwise stated. Numbers with missing values are: ethnicity, 28; born UK, 15; secure status, 24; employment status, 36; relationship status, 64; education, 13; smoking, 23; drug use, 24; alcohol use, 64; last HIV VL, 54; baseline HIV VL, 458; nadir CD4, 309; last CD4, 108.

The median age at the time of questionnaire completion was 49 [interquartile range (IQR): 47–52] years. The majority of participants were of Black African or other Black ethnicity (81.3%) with fewer women from white (12.6%) or other ethnic groups (6.1%). Four‐fifths (85.0%) of women were not born in the UK; of these, however, 95.2% had a secure immigration status (through British citizenship or right to stay documentation). The majority (88.7%) of women had completed some form of education, with more than 40% having completed university education. Two‐thirds reported some form of employment (49.3% in full‐time employment; 17.2% in part‐time employment). Just over half the women (53.0%) reported being in a current relationship (32.7% cohabiting; 20.3% non‐cohabiting).

Among the women, 177 (20.4%), 373 (43.0%) and 297 (34.2%) were pre‐menopausal, peri‐menopausal and post‐menopausal, respectively. The median (IQR) ages at questionnaire completion of women in the three groups were 47 (46–49), 49 (47–51) and 53 (50–55) years, respectively. A larger proportion of the pre‐menopausal women were born outside the UK (92.0%, 84.8% and 81.0% in the three groups, respectively), were in a cohabiting relationship (37.2%, 33.9% and 28.6%) and were in full‐time employment (51.4%, 50.4%, and 46.6%). As expected, the proportion of women with two or more comorbidities increased with later menopausal stage (4.5%, 7.5% and 12.8%), with diabetes and hypertension, in particular, occurring less commonly among pre‐ and peri‐menopausal women than those who were post‐menopausal (diabetes: 4.5%, 5.9% and 11.1%; hypertension: 16.9%, 18.0% and 25.8%).

In univariable analyses, women were less likely to be in a later menopausal stage if they were born outside of the UK [OR = 0.58 (95% confidence interval, CI: 0.40–0.82) *vs*. those born in the UK] or if they were in a relationship [non‐cohabiting OR = 0.58 (95% CI: 0.41–0.81); cohabiting OR = 0.64 (95% CI: 0.47–0.86) *vs*. not in a relationship]. By contrast, women were at greater odds of being in a later menopausal stage if they were unemployed [OR = 1.37 (95% CI: 1.03–1.83) *vs*. full‐time employment], diagnosed with one or more comorbidities [one comorbidity (OR = 1.41, 95% CI: 1.06–1.89); two comorbidities (OR = 2.04, 95% CI: 1.24–3.34); three or more comorbidities (OR = 6.31, 95% CI: 1.69–23.57) *vs*. no comorbidities] or had a baseline viral load ≥100 000 copies/mL [OR = 2.83 (95% CI: 1.34–5.98) *vs*. baseline viral load < 10 000 copies/mL].

**FIGURE 1:**
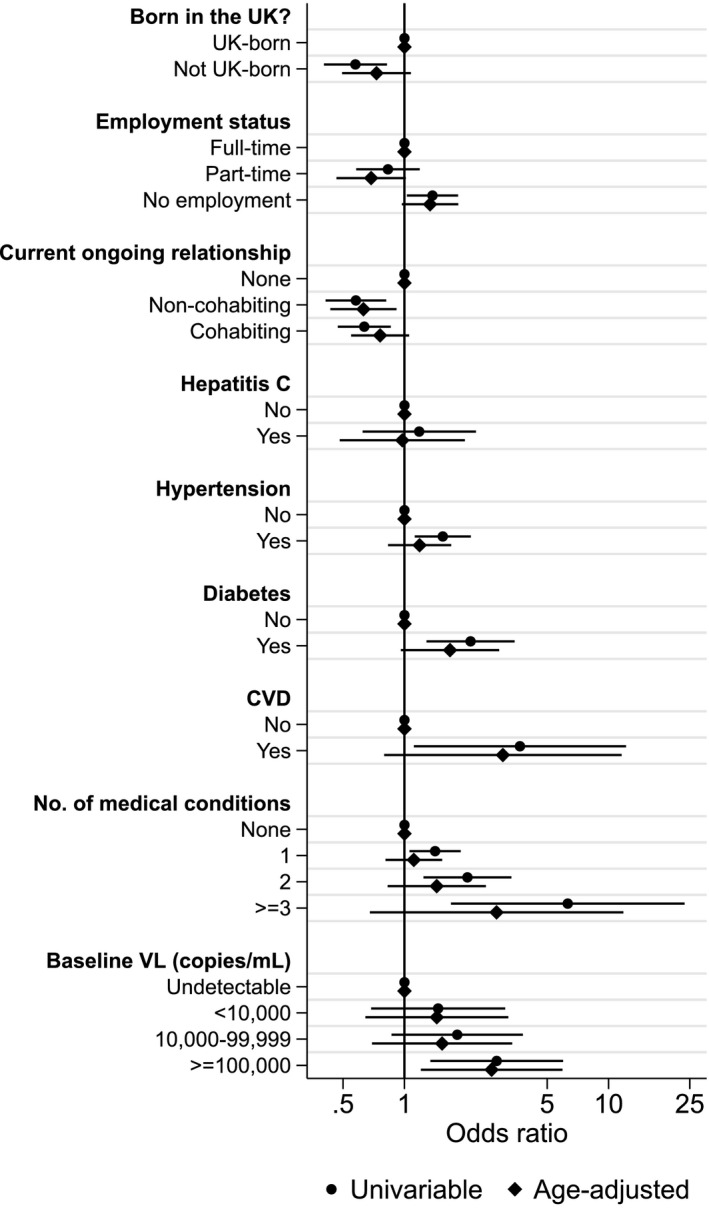
Univariable and age‐adjusted odds ratios (with 95% confidence intervals) from ordinal logistic regression exploring the association between social and clinical characteristics of women in the PRIME Study and menopausal status. CVD, cardiovascular disease; no., number; VL, viral load.

After adjusting for age, associations between menopausal status and non‐cohabiting relationship status [adjusted OR (aOR) = 0.63 (95% CI: 0.43–0.910) *vs*. not in a relationship], baseline viral load ≥ 100 000 copies/mL [aOR = 2.67 (95% CI: 1.20–5.94) *vs*. < 10 000 copies/mL] and unemployed [aOR = 1.34 (95% CI: 0.97–1.84)] remained significant (Fig. 1).

For post‐menopausal women, age at LMP ranged between 42 and 50 years, with 9.5% of women experiencing early menopause (LMP at age ≤45). Based on Kaplan–Meier estimates, the median (IQR) age at LMP was 53 (51–55) years.

Within our study population, 98.7% (836/847) responded to at least one question related to menopausal symptoms. Approximately 70% reported mild, moderate or severe menopausal symptoms (MRS > 4) and the median MRS score was 10 (IQR: 4–18; range: 0–44). Severe menopausal symptoms (MRS > 17) were reported by 27.9% (233/836) of women. Symptom burden was greater in both the peri‐ and post‐menopausal groups compared than in the pre‐menopausal group (33.3%, 31.4% and 10.3%, respectively, reported severe menopausal symptoms), with similar patterns seen for the three symptom sub‐domains (Table 2).

**TABLE 2:** Severity of menopausal symptoms reported by women who completed the Menopause Rating Scale (MRS) questionnaire stratified by menopausal status

MRS score	All	Pre‐menopausal	Peri‐menopausal	Post‐menopausal	*P*‐value
Total	836	174	369	293	
Total composite score					
None/little	243 (29.1%)	92 (52.9%)	85 (23.0%)	66 (22.5%)	< 0.001
Mild	138 (16.5%)	26 (14.9%)	61 (16.5%)	51 (17.4%)	
Moderate	222 (26.6%)	38 (21.8%)	100 (27.1%)	84 (28.7%)	
Severe	233 (27.9%)	18 (10.3%)	123 (33.3%)	92 (31.4%)	
Somatic composite score					
None/little	286 (34.2%)	103 (59.2%)	112 (30.4%)	71 (24.2%)	< 0.001
Mild	156 (18.7%)	30 (17.2%)	71 (19.2%)	55 (18.8%)	
Moderate	255 (30.5%)	34 (19.5%)	114 (30.9%)	107 (36.5%)	
Severe	139 (16.6%)	7 (4.0%)	72 (19.5%)	60 (20.5%)	
Psychological composite score					
None/little	304 (36.4%)	96 (55.2%)	109 (29.5%)	99 (33.8%)	< 0.001
Mild	145 (17.3%)	26 (14.9%)	64 (17.3%)	55 (18.8%)	
Moderate	152 (18.2%)	22 (12.6%)	75 (20.3%)	55 (18.8%)	
Severe	235 (28.1%)	30 (17.2%)	121 (32.8%)	84 (28.7%)	
Urogenital composite score					
None/little	302 (36.1%)	95 (54.6%)	111 (30.1%)	96 (32.8%)	< 0.001
Mild	100 (12.0%)	21 (12.1%)	44 (11.9%)	35 (11.9%)	
Moderate	198 (23.7%)	34 (19.5%)	95 (25.7%)	69 (23.5%)	
Severe	236 (28.2%)	24 (13.8%)	119 (32.2%)	93 (31.7%)	

MRS, Menopause Rating Scale.

Results are *n* (%).

Of the women who responded to questions related to HRT, 41.1% (329/821; 26 missing) reported that they were aware that HRT could be used to control menopausal symptoms. This proportion was considerably higher among peri‐ and post‐menopausal compared with pre‐menopausal women (42.0%, 45.7% and 26.2%, respectively; *P *= 0.001). Among the subgroup of 550 women reporting somatic symptoms, while 45.6% (246/540; 10 missing) had heard of HRT, only 8.7% (46/531; 19 missing) reported using it (currently or in the past). There was no significant association between the severity of somatic symptoms in this subgroup and either awareness (mild symptoms, 45.8%; moderate symptoms, 48.0%; severe symptoms, 40.9%; *P *= 0.63) or use (mild symptoms, 7.2%; moderate symptoms, 9.0%; severe symptoms, 9.7%; *P *= 0.74) of HRT.

Approximately 90% (745/847) of women responded to questions relating to the use of topical oestrogen. Of these, only 3.9% (29/745) reported either current or previous use. There was little evidence of an association between topical oestrogen use and menopausal status (pre‐menopausal, 2.0%; peri‐menopausal, 3.0%; post‐menopausal, 6.0%; *P *= 0.07). Only a small proportion of women experiencing any urogenital symptoms (5.6%; 27/479; 55 missing) or severe urogenital symptoms (6.6%; 14/213; 23 missing) reported using topical oestrogen.

## DISCUSSION

We found that employment status, relationship status and baseline viral load were independently associated with menopausal status in women living with HIV in the UK. Although being born in the UK, the presence of several comorbidities (specifically hepatitis C infection, hypertension, diabetes, CVD) and multi‐morbidity were associated with post‐menopausal status, these associations were attenuated after adjusting for age. Using a robust, survival analysis approach incorporating all women in the study, we estimated the median age at LMP to be 53 years. Finally, although one in five women reported severe menopausal symptoms, awareness and uptake of HRT and topical oestrogen were low.

Although menopause is defined as the cessation of menstruation, this time is usually preceded by a transition period as a result of a decline in ovarian function and circulating oestrogen. The peri‐menopausal stage can last up to 10 years and is accompanied by irregular menstruation and the onset of menopausal symptoms [11]. To our knowledge, this is the first study to assess factors that are associated with the three menopausal stages, and which therefore assesses the relationship between pre‐menopause and peri‐menopause, as well as between peri‐menopause and post‐menopause.

Married women have been reported to reach menopause at a later age [12]; however, there is much discussion around how these two factors may be linked and whether the association may be confounded by the frequency of sexual activity, parity and socio‐economic status [13]. Evidence to suggest an association between menopausal status and employment status is lacking. Individual experiences of menopause do, however, vary in terms of variety and severity, and a woman's ability to continue to work may be related to a combination of both [14]. This is the first study to report an association with baseline viral load and menopause.

Our findings suggest that reported associations of comorbidities/multi‐morbidity with menopausal status may largely reflect the older age of those with these comorbidities rather than demonstrating a direct association with menopause itself. There is evidence for increased risk of multi‐morbidity from cardiometabolic diseases, including CVD and diabetes, among women who develop premature ovarian insufficiency (age < 40 years) [15, 16]. We excluded women with premature ovarian insufficiency, and only 9.5% of post‐menopausal women in the study experienced early menopause (at 40–45 years of age), a lower proportion than reported by some other studies [17, 18, 19]. Thus, it is possible that the older age of our group may have contributed to a weakening of the association between these comorbidities and menopausal status. Although attenuated, there remained some evidence to support a higher rate of multi‐morbidity in those at later menopausal stages. It has been hypothesized that menopause‐related changes in body composition may elevate blood glucose levels, increasing the risk of diabetes, hypertension and CVD among midlife women [20], although this was not confirmed in the UK Biobank study [21]. There are currently no data on the combined impact of chronic HIV infection and menopause onset on the risk of multi‐morbidity.

We found no association between menopausal status and socio‐economic factors. In contrast to previous findings, we also found no association between menopausal status and lifestyle factors, particularly smoking, which has been well documented to be associated with early menopause in women with HIV and the general population [22, 23, 24]. However, our study includes a relatively small number of smokers and thus our ability to detect associations with smoking status may be limited. Immune function, as measured by the CD4+ T‐cell count, has also been reported to be associated with menopause among women with HIV [18, 19, 23, 25]; however, we saw no association with several HIV‐related characteristics.

We report a higher median age at LMP among women living with HIV than previous studies, using a robust approach including all women regardless of menopausal status. Use of a definition based on age at LMP is a unique approach compared with other studies among women with HIV, which more commonly report age at menopause, defined by 12 months of amenorrhoea. Many of these studies were also restricted to post‐menopausal women, with the exception of Calvet *at al*. and de Pommerol *et al*. who report much lower estimated median age at menopause (48 and 49 years, respectively) [18, 25]. On restricting analyses to the post‐menopausal group, the median age at LMP was 50 years, similar to that reported among the general population in the UK [2]. The higher age at menopause in our study may therefore reflect geographical differences and the older age distribution of women recruited to the PRIME Study compared with studies in North America (median age of 46–48 years [19, 26, 27]) and Thailand (mean age of 47 years [28]).

Previous studies exploring the impact of this have clearly shown how severe menopausal symptoms are negatively associated with HIV‐related engagement in care, adherence to treatment and, more generally, with poorer quality of life [1, 29, 30, 31, 32]. This may suggest that further research is required in understanding menopausal symptoms in the context of a woman's HIV status. The clear overlap of menopausal, HIV and ageing symptoms creates a clinical conundrum when identifying the aetiology of symptoms.

Recent analyses of prospective, longitudinal studies in the UK report that approximately 50% of post‐menopausal women in the general population have ever used HRT [33, 34]. The low rate of awareness and uptake of HRT or topical oestrogen, even among the subgroup of women who are expected to benefit from these interventions, suggest there is an unmet educational need for women with HIV and their clinicians with regard to the treatments that might help to alleviate menopausal symptoms.

Recent guidelines for the sexual and reproductive health of people living with HIV have, for the first time, introduced guidance on the management of menopause [35], recommending menopausal symptom assessment in women with HIV over the age of 45, and the use of HRT as per National Institute for Health and Care Excellence (NICE) guidelines. However, women living with HIV may encounter challenges when seeking menopause care. A study across primary care clinicians in the UK (the setting in which most women with menopausal symptoms will be managed) revealed that there was little confidence amongst primary care practitioners when managing menopausal symptoms in women with HIV [36].

The PRIME Study is a large, representative sample of women accessing HIV care in the UK linking to clinical data and using a range of validated tools. However, there are some limitations. Age at LMP of post‐menopausal women was estimated, and therefore, although these are the only data available in the UK, we acknowledge this will be an imprecise estimate. In addition, we had limited or no data on factors including change in body mass index, age at menarche, and age at first birth which could result in residual confounding. Although transgender men were not specifically excluded from the study, the use of hormonal medications may have resulted in the exclusion of some transgender men. As influences on menopause experience are likely to differ in this group, our findings may have limited generalizability outside cisgender women. Finally, the cross‐sectional nature of the study does not allow us to determine the true sequence of events, and therefore results must be interpreted with caution.

In summary, our findings from a large, representative cohort of women accessing HIV care in the UK contribute to a much‐needed evidence base for the experience of the population of women with HIV who are ageing and increasingly undergoing menopause. Awareness and uptake of HRT and topical oestrogen were low, even in the presence of significant symptoms. The publication of national guidelines on HIV and menopause are an important step forwards, but continued education of healthcare providers in both specialist and primary care is required to ensure that these guidelines are translated into improvements in care. Finally, we welcome the development of patient resources and support tailored to older women living with HIV [37], empowering them to maximize their own health and well‐being as they reach midlife and beyond.

## CONFLICTS OF INTEREST

None to declare.

## AUTHOR CONTRIBUTIONS

HO: first author, design of work, analysis, interpretation, manuscript writing and corresponding author. KH, LS and RD: design of work, writing and technical editing of manuscript. FB, RG, FP, JR, NM, AS, JS, AT, RJ, JF, and MR: revision of manuscript. CAS and ST: statistical analysis, writing and technical editing of manuscript, final overseeing of manuscript submission. Authors agreed on all aspects of the work for the final manuscript.

## Appendix 1

Symptoms reported by the Menopause Rating Scale:

- Somatic symptoms: episodes of hot flushes/sweating, heart discomfort, sleeping disorders and joint/muscle complaints
- Psychological symptoms: depression, irritability, anxiety and exhaustion
- Urogenital symptoms: sexual problems, vaginal dryness and urinary complaints

Composite scores were generated by summing the score of individual symptoms in each domain. Each composite score was separated into four groups defining the severity of symptoms as outlined below:

- Somatic symptoms: none/little, 0–2; mild, 3–4; moderate, 5–8; severe, 9+
- Psychological symptoms: none/little, 0–1; mild, 2–3; moderate, 4–6; severe, 7+
- Urogenital symptoms: none/little, 0; mild, 1; moderate, 2–3; severe, 4+
- Total composite score: none/little, 0–4; mild, 5–8; moderate, 9–16; severe, 17+
